# Supplementary material for: Dental emergency: Scoping review
Source: PLoS One. 2020 Feb 14;15(2):e0222248. doi: 10.1371/journal.pone.0222248 (PMC7063673; doi:10.1371/journal.pone.0222248)
Supplement: S3 Fig — (PDF) [file pone.0222248.s003.pdf]

S3. Fig 3. Gaps in dental emergency research
