# Supplementary material for: Dental emergency: Scoping review
Source: PLoS One. 2020 Feb 14;15(2):e0222248. doi: 10.1371/journal.pone.0222248 (PMC7063673; doi:10.1371/journal.pone.0222248)
Supplement: Attachment — Submitted filename: Response to reviewers.docx [file pone.0222248.s006.docx]

December 02, 2019

Marta Pascual
Academic Editor
PLOS ONE

PONE-D-19-23071
Dental emergency: scoping review

Dear Dra. Pascual:

Thank you very much for reviewing our manuscript. We also greatly appreciate the reviewers for their complimentary comments and suggestions. We have carried out the experiments that the reviewers suggested and revised the manuscript accordingly PLOS orientations.

Please find attached a point-by-point response to academic editor’s and reviewer’s concerns. We hope that you find our responses satisfactory and that the manuscript is now acceptable for publication.

Sincerely,

Karla Frichembruder, Ph.D

Researcher

Centro de Pesquisas em Odontologia Social

Faculdade de Odontologia UFRGS

Porto Alegre, RS, Brazil

Email: kfrichem@gmail.com

We appreciate that the academic editor, editorial staff and reviewer’s comments. The followings are our point-by-point responses.

**Academic Editor Comments to the Author**

Comment: “To enhance the reproducibility of your results, we recommend that if applicable you deposit your laboratory protocols in [protocols.io](http://protocols.io/), where a protocol can be assigned its own identifier (DOI) such that it can be cited independently in the future.”

Response: the protocol was deposited in [protocols.io](http://protocols.io/) and registered as: [dx.doi.org/10.17504/protocols.io.8nshvee](http://dx.doi.org/10.17504/protocols.io.8nshvee)

Comment: “Editorial staff concerns about the grammar, usage, and overall readability of the manuscript.”

Response: the manuscript was edited and certificated by American Journal Experts on November 9, 2019. The verification code is 77B4-1CB3-2CED-A848-E089. A marked-up copy of the manuscript was uploaded with highlights changes made to the original version (yellow=original and green=changes).

**Review Comments to the Author
Reviewer #1**

Comment: “More search engines are recommended, as you stated: The search was carried out in the Medline (PubMed), Embase and Web of Science databases.”

Response: an additional search was carried out using Scopus, resulting in one new article that was added to the results. There were no changes in the final model and research gaps sections. Comment: “More statistical analysis is required to present your results clearly.”

Response: only descriptive analyses were provided because scoping reviews do not make other statistics analyses. Data synthesis by metarregression is a common feature of Systematic Reviews with meta-analysis, but this was not the case of the present study, as outcomes of individual studies were not similar enough to allow data synthesis. Others scoping reviews published in PLOS ONE used the same method: <https://journals.plos.org/plosone/article?id=10.1371/journal.pone.0215278>, <https://journals.plos.org/plosone/article?id=10.1371/journal.pone.0156376>).

A marked-up copy of the manuscript was uploaded with highlights changes made to the original version (yellow=original and green=changes).

**Reviewer #3**

Comment: “In general, the manuscript followed a clear and well written format that was easy to follow and understand. However, there are minor grammatical errors which I have highlighted in the attached document along with this form.”

Response: we have corrected grammatical mistakes and the manuscript was revised by American Journal Experts.

A marked-up copy of the manuscript was uploaded with highlights changes made to the original version (yellow=original and green=changes).
